# Increased serum extrachromosomal circular DNA SORBS1^circle^ level is associated with insulin resistance in patients with newly diagnosed type 2 diabetes mellitus

**DOI:** 10.1186/s11658-023-00530-0

**Published:** 2024-01-12

**Authors:** Xiang Kong, Shu-jun Wan, Tian-bing Chen, Lan Jiang, Yu-jie Xing, Ya-ping Bai, Qiang Hua, Xin-ming Yao, Yong-li Zhao, Hong-mei Zhang, De-guo Wang, Qing Su, Kun Lv

**Affiliations:** 1 Anhui Provincial Key Laboratory of Non-Coding RNA Basic and Clinical Transformation, Wannan Medical College, Wuhu, 241002 China; 2 Geriatric Endocrinology Unit, Department of Gerontology, The First Affiliated Hospital of Wannan Medical College, Yijishan Hospital, Wuhu, 241001 China; 3 Central Laboratory of Yijishan Hospital, Wuhu, 241001 China; 4 Department of Endocrinology, The First Affiliated Hospital of Wannan Medical College, Yijishan Hospital, Wuhu, 241001 China; 5 Department of Endocrinology, Xinhua Hospital, Shanghai Jiaotong University School of Medicine, Shanghai, 200092 China

**Keywords:** Extrachromosomal circular DNAs, T2DM, SORBS1, Insulin resistance

## Abstract

**Background:**

Extrachromosomal circular DNAs (eccDNAs) exist in human blood and somatic cells, and are essential for oncogene plasticity and drug resistance. However, the presence and impact of eccDNAs in type 2 diabetes mellitus (T2DM) remains inadequately understood.

**Methods:**

We purified and sequenced the serum eccDNAs obtained from newly diagnosed T2DM patients and normal control (NC) subjects using Circle-sequencing. We validated the level of a novel circulating eccDNA named sorbin and SH3‐domain‐ containing‐1^circle97206791–97208025^ (SORBS1^circle^) in 106 newly diagnosed T2DM patients. The relationship between eccDNA SORBS1^circle^ and clinical data was analyzed. Furthermore, we explored the source and expression level of eccDNA SORBS1^circle^ in the high glucose and palmitate (HG/PA)-induced hepatocyte (HepG2 cell) insulin resistance model.

**Results:**

A total of 22,543 and 19,195 eccDNAs were found in serum samples obtained from newly diagnosed T2DM patients and NC subjects, respectively. The T2DM patients had a greater distribution of eccDNA on chromosomes 1, 14, 16, 17, 18, 19, 20 and X. Additionally, 598 serum eccDNAs were found to be upregulated, while 856 eccDNAs were downregulated in T2DM patients compared with NC subjects. KEGG analysis demonstrated that the genes carried by eccDNAs were mainly associated with insulin resistance. Moreover, it was validated that the eccDNA SORBS1^circle^ was significantly increased in serum of newly diagnosed T2DM patients (106 T2DM patients vs. 40 NC subjects). The serum eccDNA SORBS1^circle^ content was positively correlated with the levels of glycosylated hemoglobin A1C (HbA1C) and homeostasis model assessment of insulin resistance (HOMA-IR) in T2DM patients. Intracellular eccDNA SORBS1^circle^ expression was significantly enhanced in the high glucose and palmitate (HG/PA)-induced hepatocyte (HepG2 cell) insulin resistance model. Moreover, the upregulation of eccDNA SORBS1^circle^ in the HG/PA-treated HepG2 cells was dependent on generation of apoptotic DNA fragmentation.

**Conclusions:**

These results provide a preliminary understanding of the circulating eccDNA patterns at the early stage of T2DM and suggest that eccDNA SORBS1^circle^ may be involved in the development of insulin resistance.

**Supplementary Information:**

The online version contains supplementary material available at 10.1186/s11658-023-00530-0.

## Background

A group of novel circular DNAs referred as extrachromosomal circular DNAs (eccDNAs), which is physically derived from genomic DNA, has been identified in tissues, plasma, serum and urine by virtue of new technologies such as whole-genome sequencing, ATAC-sequencing and Circle-sequencing [1–3]. eccDNAs contribute to oncogene plasticity [4–6] and drug resistance [7, 8]. Wang et al. demonstrated that eccDNAs exhibit high innate immunostimulatory activity [9]. Recently, the characteristics of eccDNAs in peripheral blood mononuclear cells of chronic kidney disease patients have been reported [10].

Type 2 diabetes mellitus (T2DM) is a serious, complex chronic condition characterized by hyperglycaemia and insulin resistance. Significant variations in the concentrations of cell-free DNA and mitochondrial DNA have been validated in the serum or plasma of of patients with T2DM compared to normal control (NC) subjects. [11–13]. However, there is currently a lack of published data regarding eccDNAs in T2DM patients.

In the present study, we have characterized serum eccDNAs in newly diagnosed T2DM patients and NC subjects, and identified sorbin and SH3‐domain‐ containing‐1^circle 97206791–97208025^ (designated as SORBS1^circle^) as a novel eccDNA. SORBS1^circle^ has been validated as the most significantly upregulated eccDNA in the serum of T2DM patients. Furthermore, we determined the clinical value of eccDNA SORBS1^circle^ in a cohort of newly diagnosed T2DM patients and found that the increase of eccDNA SORBS1^circle^ is associated with insulin resistance. Finally, we have demonstrated that eccDNA SORBS1^circle^, generated from apoptotic DNA fragmentation, was significantly enhanced in the high glucose and palmitate (HG/PA)-induced HepG2 cell insulin resistance model.

## Methods

### Participants

This study was approved by the Research Ethics Committee of the Yijishan Hospital (approval number: 202022) in accordance to the requirement of the Helsinki Declaration. This prospective study was clinically registered (Clinical trial number: ISRCTN12415732, https://www.isrctn.com/). A total of 146 subjects including 106 newly diagnosed T2DM patients and 40 age-matched and hospital-based NC subjects were enrolled from September 2020 to October 2021 in this study. Patients with T2DM were diagnosed according to the 2019 Standards of American Diabetes Association [14]. "Newly diagnosed T2DM" refers to patients who have been recently identified with type 2 diabetes and have not yet received any treatment. Exclusion criteria for patient selection were as follows: (1) with type 1 diabetes or any other type of diabetes; (2) with diabetic acute complications; (3) with inflammatory illnesses; (4) with kidney or liver diseases; (5) with autoimmune diseases; (6) with malignant tumors. The hospital-based NC subjects were strictly evaluated by physicians. Participants' health status was assessed through a physical examination, vital signs monitoring, and clinical laboratory tests, which included hematology, biochemistry, coagulation, and urinalysis. The NC subjects had no history or diagnosis of chronic diseases and were not currently taking any medications. Pregnant and nursing women were excluded from the study.

### Biochemical analysis

Serum fasting blood glucose (FBG), 2-h postprandial blood glucose (2 hPBG), total cholesterol (TC), triglycerides (TG), high density lipoprotein cholesterol (HDL-C), low density lipoprotein cholesterol (LDL-C), alanine transaminase (ALT), aspartate transaminase (AST), creatinine (Cr) and uric acid (UA) levels were examined using enzymatic colorimetric methods on a Hitachi 7600 analyzer (Hitachi, Japan). Fasting insulin (FIns) and 2-h postprandial insulin (2 hPIns) concentration were measured using an ELISA kit (Abcam, Cambridge, UK). Glycosylated hemoglobin A1C (HbA1C) level was determined by an automatic glycohemoglobin analyzer (ADAMS A1c HA-8180, ARKRAY, Japan). Insulin resistance was evaluated using the homeostasis model HOMA-IR, which was calculated by using the following formula: FIns × FBG/22.5 [15]. Islet β-cell function was evaluated using the HOMA-β, which was calculated by using the following formula: 20 × FIns/(FBG-3.5) [15].

### Circle sequencing

Circle-sequencing (Circle-seq) was employed to identify serum eccDNAs in four patients with newly diagnosed T2DM (36.75 ± 9.5 years; two males, two females) and four age- and sex-matched NC subjects (35.25 ± 4.6 years; two males, two females). The eccDNA Circle-seq service was provided by CloudSeq Biotech Inc. (Shanghai, China). The Circle-seq data have been deposited into the Genome Sequence Archive (GSA, https://ngdc.cncb.ac.cn/gsa/) with accession number HRA002618.

Peripheral venous blood (5 ml) was collected using vacutainer serum tubes (GD050SG, Gongdong™ Medical, Zhejiang, China) from participants fasting at least 12 h, and the serum was immediately separated by a 10 min centrifugation at 1600 g. The serum samples were stored at -80 °C until use. Serum DNA extractions were performed using QIAamp Circulating Nucleic Acid Kit (Qiagen, Germany). For elimination of linear DNA and enrichment of eccDNA, 25 ng of serum DNA were treated with 5 units of exonuclease V (New England Biolabs, USA) in a 50 μl reaction system at 37 °C for 5 min, followed by column purification using MinElute Reaction Cleanup Kit (Qiagen, Germany). eccDNA enriched from 25 ng of serum DNA were processed using NEBNext® Ultra™ DNA Library Prep Kit (New England Biolabs, USA). DNA libraries were sequenced on Illumina NovaSeq 6000 with 150 bp paired end mode according to the manufacturer’s instructions.

Paired-end reads were harvested from Illumina NovaSeq 6000 sequencer. After 3’ adaptor-trimming and low quality reads removing by cutadapt software (v 1.9.1), the high quality clean reads were aligned to the reference genome (UCSC hg19) with Bwa software (v 0.7.12). Then, Circle-Map software (v 1.1.4) was used to detect eccDNA within all samples, and Samtool software (v 0.2) was used to get raw soft-clipped read counts of the break point. The EdgeR software (v0.6.9) was then employed to normalize and identify differentially expressed eccDNAs filter by *p*-value and |log2FC| value (fold change). Bedtools software (v 2.27.1) was used to annotate the eccDNAs.

### Validation and KEGG analysis

Quantitative PCR (qPCR) was performed with the outward facing primers (Table 1) using SYBR Green PCR Master Mix (Thermo Fisher, USA) in Applied Biosystems Quantstudio 5 Real-Time PCR System (Thermo Fisher, USA). For eccDNA validation, the pGEX-5X-2 plasmid (1.2 × 10^6^ copy/ml) was added to the samples prior to eccDNA extraction and purification, and used as an internal control [1, 5]. The eccDNA detection for each sample was performed using triplicate wells, and the results were averaged. The CT values of the target eccDNA and PGEX-5X-2 were calculated and the relative expression levels of eccDNA were analyzed using the 2^−ΔΔCt^ method. The detailed steps of qPCR were provided in the Additional file 1. Furthermore, Sanger sequencing of PCR products was conducted using an automatic genetic analyzer (ABI 3730xl, Applied Biosystems, USA). This sequencing method was utilized to confirm the loop and genome position of the target eccDNA. The upregulated eccDNAs carrying full or partial genes were extracted and analyzed with KEGG (http://www.genome.jp/kegg/) pathway enrichment analysis.

**Table 1:** Sequences of outward facing primers

	Genomic position	Sequence	Amplicon length (bp)
eccDNA SORBS1^circle^	chr10:97206791–97208025	F: ATTTACACTGGGCCATGAGG	142
		R: AGTGATTCTCCTGCCTCTGC	
eccDNA DGKI^circle^	chr7:137407136–137407412	F: TTTGTCATTGCCACCTCAAC	134
		R: GTAATGCTGGCTCGCAGTTT	
eccDNA COG2^circle^	chr1:230789246–230789354	F: AATTCTACTAGCCAGAGTGTTTAGA	51
		R: TCTGGCTGGGCACAGTGG	
eccDNA FAM20C^circle^	chr7:217309–217415	F: CAGACTGGCTGTGGGTGAG	75
		R: AGGAGCCAGGGATGATGAC	
eccDNA ETV1^circle^	chr7:14009902–14010102	F: AACTTGGGCGAGAAGGTATG	131
		R: GTTGCTGCTTTGTGTGTGTG	
pGEX-5X-2		F: GGGCTGGCAAGCCACGTTTGGTG	176
		R: CCGGGAGCTGCATGTGTCAGAGG	

*F* Forward, *R* Reverse, *DGKI* diacylglycerol kinase iota, *COG2* conserved oligomeric Golgi complex subunit 2, *FAM20C* family with sequence similarity 20- member C, *ETV1* ETS variant 1

### Cell culture and treatment

Human hepatoma HepG2 cells (Procell, Cat. No.: CL-0103, Wuhan, China) were cultured in low-glucose DMEM (5.5 mM, Gibco, USA) supplemented with 10% fetal bovine serum (FBS, Gibco, USA). Following the incubation of HepG2 cells in complete medium for 24 h, they were segregated at a density of 5.0 × 10^5^ cells/well in 6-well plates. HepG2 cells were stimulated with HG/PA (25.0 mM glucose and 0.5 mM palmitate) for 12, 24 or 36 h. Some HepG2 cells were treated with 100 nM insulin for 20 min before being harvested.

### Western blot analysis

HepG2 cells were washed twice with cold PBS and then lysed in 100 μl of RIPA lysis buffer (Beyotime, China). The proteins obtained were separated by SDS-PAGE and immediately transferred onto nitrocellulose membranes. The membranes were incubated with primary antibodies overnight and then with the appropriate secondary antibodies for 2 h. The antibodies used were phosphorylated protein kinase B (P-Akt, CST, USA), total Akt (T-Akt, CST, USA), Bcl-2 (Abclone, China) and Bax (Abclone, China). Data were analyzed using the ImageJ software.

### Measure of hepatocytes eccDNA SORBS1^circle^

eccDNAs were isolated from the treated HepG2 cells using a HiSpeed midi-prep DNA isolation kit (Qiagen, Germany). The linear DNA was digested using 30 units of Plasmid-Safe ATP-dependent DNase (Epicentre, USA) and incubated at 37 °C overnight for 12 h. The remaining circular DNA was purified using DNA Clean & Concentrator-5 Kit (ZYMO, USA) [16]. Intracellular eccDNA SORBS1^circle^ expression was measured by qPCR as mentioned above.

### FISH assay of eccDNA SORBS1^circle^

Fluorescence in situ hybridization (FISH) assay was performed in HepG2 cells using fluorescent in situ hybridization kit (Ribobio, China) as manufacturer’s instruction. Cy3-labeled probe specific to the junction sites of SORBS1^circle^ was designed at RiboBio. Briefly, HepG2 cells were first fixed with 4% paraformaldehyde for 10 min in room temperature and treated with 0.5% Triton X-100 for 20 min in 4 ℃. After washing with PBS buffer three times, cells were treated with pre-hybridization buffer at 37 ℃ for 30 min, and then incubated with hybridization buffer containing 500 nM FISH probes to SORBS1^circle^ at 37 ℃ overnight. After incubation, cells were washed with 4 × saline sodium citrate (SSC, Beyotime, China) with 0.1% Tween-20 three times at 42 ℃, following with 2 × SSC, 1 × SSC at 42 ℃ and then PBS once at room temperature. Nuclei was labeled with DAPI. The representative images were captured using a confocal microscope.

### DNA fragmentation assay

HepG2 cells were cultured with 6-well plates and stimulated with HG/PA for 36 h in the presence or absence of 100 μM Z-VAD-FMK (an apoptosis inhibitor, Sellect, China). DNA fragmentation was measured using the Apoptotic DNA Ladder Isolation Kit (Abcam, USA). We standardized DNA concentration for each sample by counting the cells for each group. Briefly, approximately 2 × 10^6^ cells for each group were collected by centrifugation for 5 min at 500 g. Then, cell pellets were suspended with 100 µl DNA Ladder Extraction Buffer and centrifuged at 1600 g for 5 min. The cell supernatants were treated with 5 µl Enzyme A Solution at 37 °C for 10 min, and then incubated with 5 µl Enzyme B Solution at 50 °C for 30 min. Then, 5 µl ammonium acetate solution and 100 µl isopropanol were added into each sample, mixed well and kept at -20 °C for 10 min. Following the precipitation and purification of the DNA, samples were resuspended in 30 µl in DNA Suspension Buffer and loaded the same volume on 1.5% agarose gel stained with ethidium bromide and visualized under UV light. In the process, we maintained the assay in the same conditions for all experiments and repeated it three times.

### Statistical analysis

Data are presented as the mean ± standard deviation (SD). Between-group comparisons were tested by two-tailed paired Student’s t-test or Chi-squared test. The differences among groups were determined by the use of one-way analysis of variance followed by the Tukey’s multiple comparisons test. Relationship between serum eccDNA SORBS1^circle^ level and other clinical and biochemical parameters was analyzed using Pearson correlation analysis. Multiple stepwise linear regression analysis was performed to identify the factors affecting the change in serum eccDNA SORBS1^circle^ level. Values of *P* < 0.05 were considered statistically significance.

## Results

### Detection of eccDNAs in serum of newly diagnosed T2DM patients by Circle-seq

**Fig. 1:**
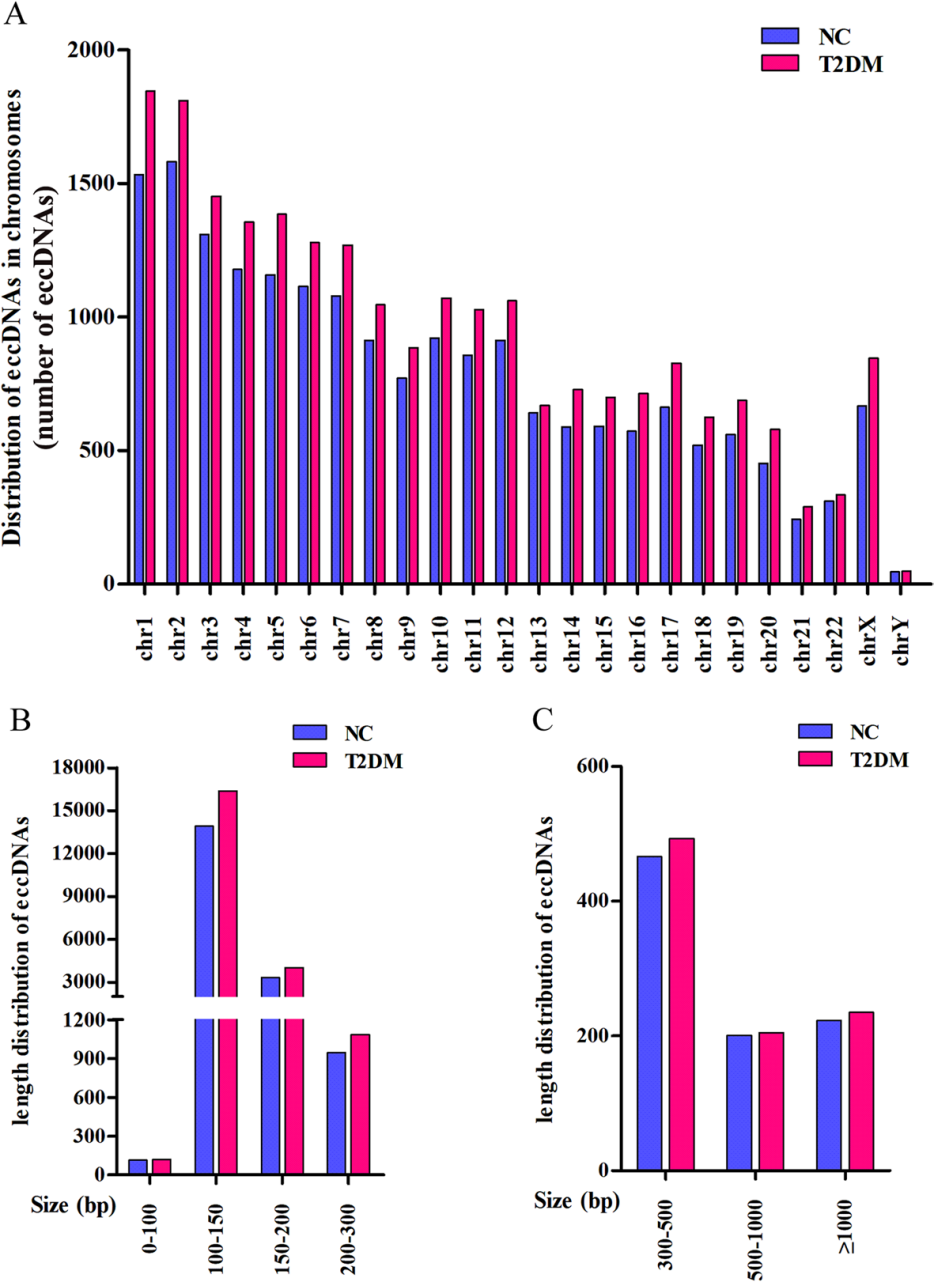
Features of eccDNAs detected in serum of newly diagnosed T2DM patients and NC subjects. **A** The distribution of eccDNAs in different chromosomes. **B**, **C** The length density distribution of eccDNAs

To detect eccDNAs from similar pair regions of the human genome, we used the Circle-Map software under the screening condition with split reads ≥ 1. As a result, 22,543 and 19,195 eccDNAs were found in serum samples obtained from newly diagnosed T2DM patients and NC subjects, respectively. The average amount of serum eccDNAs in T2DM patients was higher than that in NC subjects (8090 ± 904 *vs.* 6462 ± 879, *P* < 0.05). These eccDNAs were derived from any chromosome, especially in chromosome 1 and 2. The T2DM patients had a greater distribution of eccDNAs on chromosomes 1, 14, 16, 17, 18, 19, 20 and X (increase by over 20% vs. NC subjects, Fig. 1A and Additional file 1: Table S1). The length of serum eccDNAs varied greatly but majority of eccDNAs fragments ranged between 100 and 150 bp (Fig. 1B, C).

### eccDNAs expression analysis and validation

**Fig. 2:**
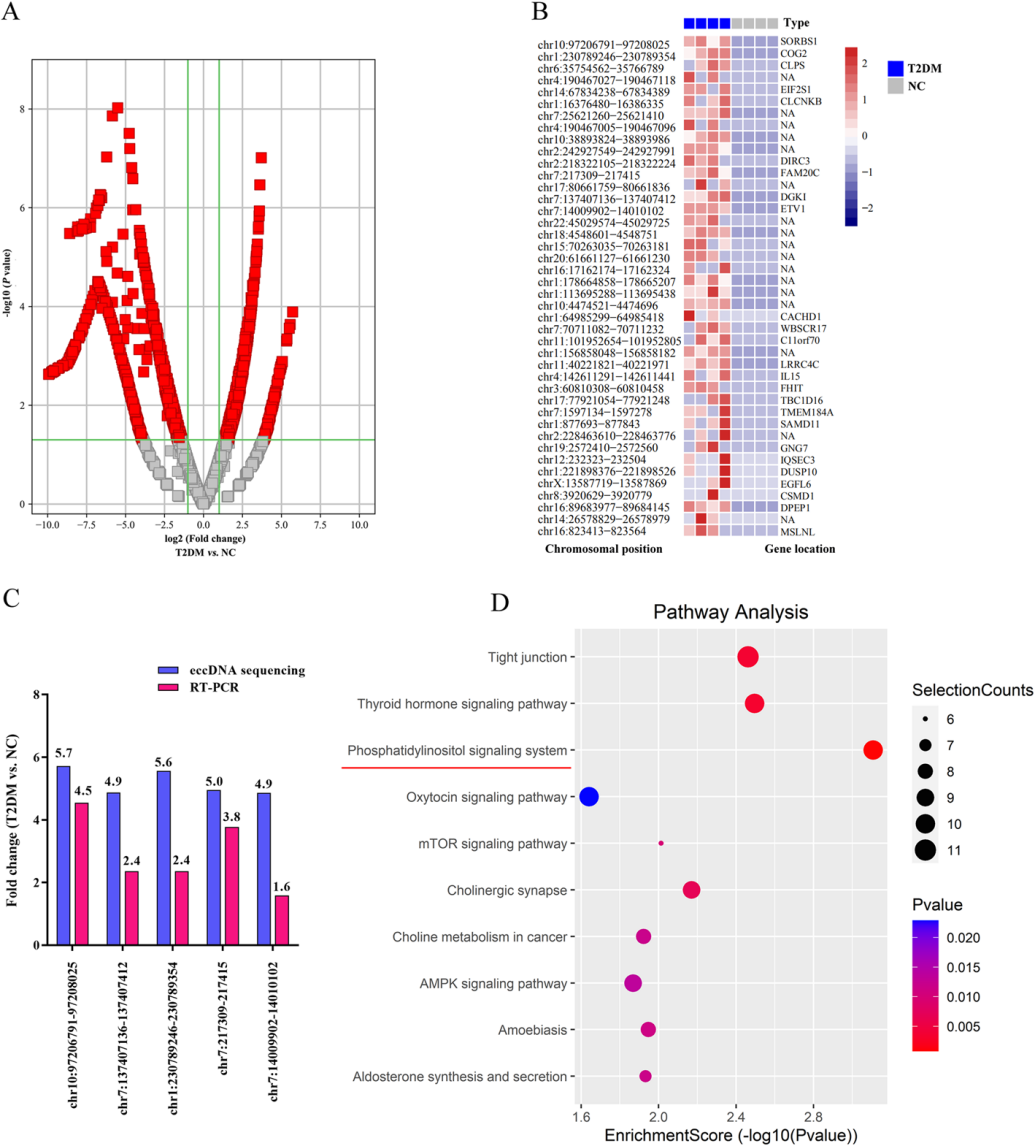
Profile of Circle-seq data for eccDNAs. **A** The volcano plot of differentially expressed eccDNA in the serum of newly diagnosed T2DM patients and NC subjects. **B** The top 42 upregulated circulating eccDNAs in T2DM patients compared with NC subjects. **C** Five upregulated eccDNAs were selected to validate using qPCR with the outward facing primers. **D** The 10 most enriched KEGG pathways in T2DM patients based on eccDNAs carrying genes

According to the expression profiling data, 1454 obviously dysregulated serum eccDNAs were identified with a set filter (fold change ≥ 2.0, *P* < 0.05) in newly diagnosed T2DM patients compared with NC subjects: 598 were upregulated, while 856 were downregulated (Fig. 2A). The top 42 upregulated eccDNAs in T2DM patients compared with NC subjects were revealed in Fig. 2B and Additional file 1: Table S2. Five upregulated eccDNAs were randomly selected for the validation of the eccDNA sequencing data using qPCR. As shown in Fig. 2C, the PCR results indicated an elevation in the expression levels of these five eccDNAs in the serum of newly diagnosed T2DM patients, with a similar trend as observed in the eccDNAs sequencing data. To further explore the potential functions of upregulated eccDNAs in T2DM patients, a pathway analysis of the genes carried by eccDNAs was employed using KEGG. Compared with the NC subjects, genes on eccDNAs in T2DM patients had particular enrichments in the phosphatidylinositol signaling system (Fig. 2D), which plays an important role in insulin resistance.

### Serum eccDNA SORBS1^circle^ level is increased in newly diagnosed T2DM patients

**Fig. 3:**
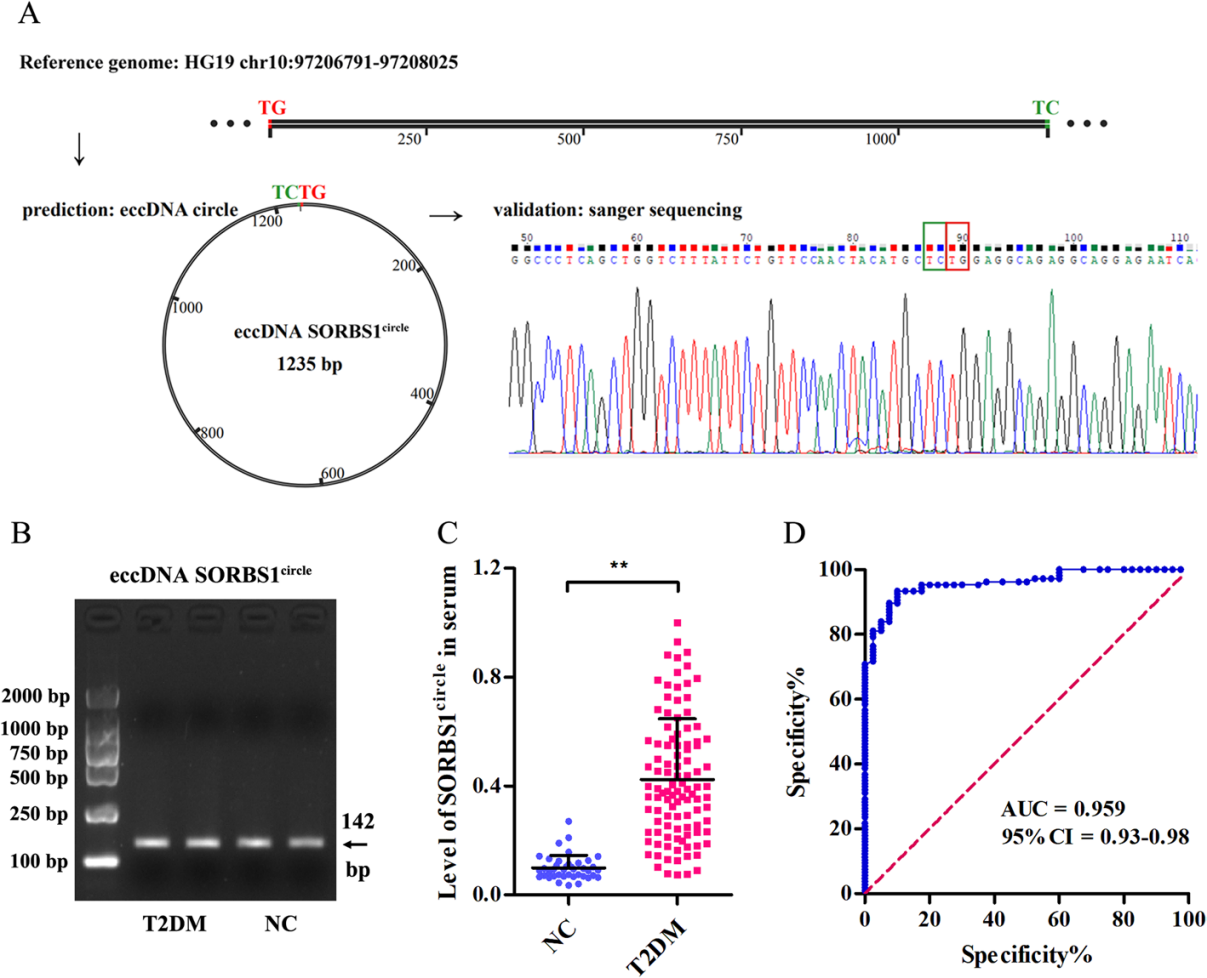
Serum eccDNA SORBS1^circle^ level is increased in newly diagnosed T2DM patients. **A** Visualization of the most significantly upregulated serum eccDNA SORBS1^circle^. The junction site of eccDNA SORBS1^circle^ was TCTG. **B** Gel electrophoresis images of validated serum eccDNA SORBS1^circle^. **C** qPCR validation of serum eccDNA SORBS1^circle^ level in serum obtained from 106 newly diagnosed T2DM patients and 40 NC subjects. **P < 0.01. **D** ROC curves for the capacity of the serum eccDNA SORBS1^circle^ to differentiate T2DM patients from NC subjects

We selected the markedly upregulated eccDNA which named as SORBS1^circle 97206791–97208025^ by its chromosomal origin SORBS1 for further investigation. Sanger sequencing confirmed the circular structure and junction site of eccDNA SORBS1^circle^ (Fig. 3A). Gel electrophoresis images of eccDNA SORBS1^circle^ was shown in Fig. 3B. We next validated eccDNA SORBS1^circle^ level in serum obtained from 106 newly diagnosed T2DM patients and 40 NC subjects, and confirmed its content was significantly increased in T2DM patients (fold change: 4.27, Fig. 3C). Moreover, we evaluated the diagnostic value of serum eccDNA SORBS1^circle^ level and found that the area under ROC curve (AUC) was 0.959 (95% CI 0.93–0.98), sensitivity was 88.0% and specificity was 92.0% for the patients with newly diagnosed T2DM and NC subjects (Fig. 3D).

### Serum eccDNA SORBS1^circle^ level is associated with insulin resistance in newly diagnosed T2DM patients

Baseline clinical and biochemical characteristics of all study subjects were shown in Additional file 1: Table S3. As shown in Table 2, correlation analysis revealed that eccDNA SORBS1^circle^ level was positively correlated with serum content of FBG (*R* = 0.351, *p* < 0.01), HbA1c (*R* = 0.257, *p* < 0.01), FIns (*R* = 0.629, *p* < 0.01) and HOMA-IR (*R* = 0.751, *p* < 0.01). In a multiple regression model with serum eccDNA SORBS1^circle^ level as a dependent variable and FBG, 2 hPBG, HbA1c, FIns, HOMA-IR and HOMA-β as independent variables, HbA1c and HOMA-IR were the independent predictors of eccDNA SORBS1^circle^ content (Table 2). In another model, we added all clinical and biochemical factors as independent variables and the results also suggested that HbA1c and HOMA-IR were independently and significantly associated with serum eccDNA SORBS1^circle^ (data not shown).

**Table 2:** Correlation and regression analysis between serum eccDNA SORBS1^circle^ level and other clinical and biochemical parameters

Varibles	Pearson correlation		Multiple linear regression		
	*R* value	*P* value	*β* value	*T* value	*P* value
Age	− 0.015	0.880			
Sex	− 0.069	0.484			
BMI	0.123	0.211			
SBP	0.014	0.888			
DBP	− 0.011	0.913			
FBG	0.351	0.000**	0.079	1.124	0.264
2 hPBG	0.177	0.069	0.109	1.692	0.094
HbA1c	0.257	0.008**	0.141	2.193	0.031*
FIns	0.629	0.000**	− 0.069	− 0.544	0.588
2 hPIns	0.059	0.548			
HOMA-IR	0.751	0.000**	0.751	11.593	0.000**
HOMA-β	0.177	0.069	− 0.067	− 0.982	0.329
TC	0.073	0.458			
TG	0.106	0.278			
HDL-C	− 0.091	0.354			
LDL-C	− 0.003	0.978			
ALT	0.005	0.962			
AST	− 0.023	0.819			
Cr	− 0.025	0.798			
UA	− 0.153	0.117			

*P < 0.05, **P < 0.01. *BMI* Body mass index, *SBP* systolic blood pressure, *DBP* diastolic blood pressure, *FBG* fasting blood glucose, *2 hPBG* 2-h postprandial blood glucose, *HbA1c* glycosylated hemoglobin A1c, *FIns* fasting insulin, *2 hPIns* 2-h postprandial insulin, *HOMA-IR* homeostasis model assessment of insulin resistance, *HOMA-β* homeostasis model assessment of islet β-cell function, *TC* total cholesterol, *TG* triglycerides, *HDL-C* high-density lipoprotein cholesterol, *LDL-C* low-density lipoprotein cholesterol, *ALT* alanine transaminase, *AST* aspartate transaminase, *Cr* creatinine, *UA* uric acid

### eccDNA SORBS1^circle^ level is increased in HG/PA-treated HepG2 cells

**Fig. 4:**
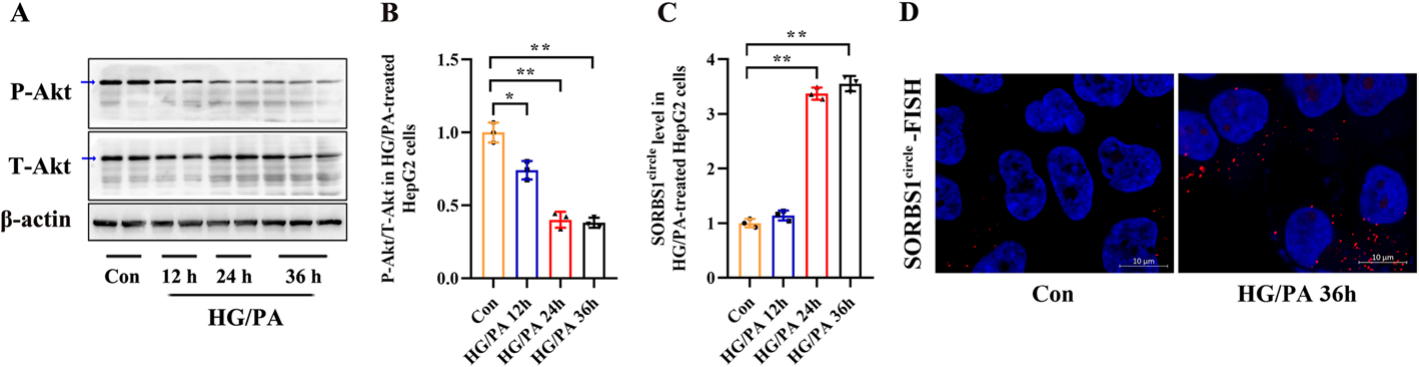
Expression of eccDNA SORBS1^circle^ in HG/PA-treated HepG2 cells. **A**, **B** The protein levels of P-Akt S473 and T-AKT in HepG2 cells treated with 25.0 mM glucose (HG) and 0.5 mM palmitate (PA) were evaluated by western blotting. These HepG2 cells were treated with 100 nM insulin for 20 min before being harvested. **C** qPCR was performed for the determination of intracellular eccDNA SORBS1^circle^ level in HG/PA-treated HepG2 cells. **D** Visualization of eccDNA SORBS1^circle^ expression in control and HG/PA-treated HepG2 cells using FISH assay. The complete image of FISH assay was shown in Additional file 1: Fig. S1. Bar = 10 μm. Data are presented as the mean ± SD of three independent experiments. *P < 0.05, **P < 0.01

Insulin resistance is a major pathophysiological event in the pathogenesis of T2DM. Insulin resistance with impaired glucose metabolism appears frequently in liver tissue during the development of T2DM [17]. Akt is a key signaling molecule involved in the insulin signaling pathway. In cases of insulin resistance, the phosphorylation of Akt is impaired [18]. Therefore, we measured the expression of eccDNA SORBS1^circle^ in an HG/PA-induced HepG2 cell insulin resistance model, characterized by a significantly decreased P-Akt/T-Akt ratio (Fig. 4A, B). Intracellular eccDNA SORBS1^circle^ level measured by qPCR assay was significantly increased in HepG2 cells treated with HG/PA for 24 or 36 h (Fig. 4C). A FISH assay was performed to detect eccDNA SORBS1^circle^ using a Cy3-labeled probe. The results showed that the level of eccDNA SORBS1^circle^ was increased in HepG2 cells after HG/PA treatment for 36 h (Fig. 4D and Additional file 1: Fig. S1).

### Increased eccDNA SORBS1^circle^ level in HG/PA-treated HepG2 cells is associated with apoptotic DNA fragmentation

**Fig. 5:**
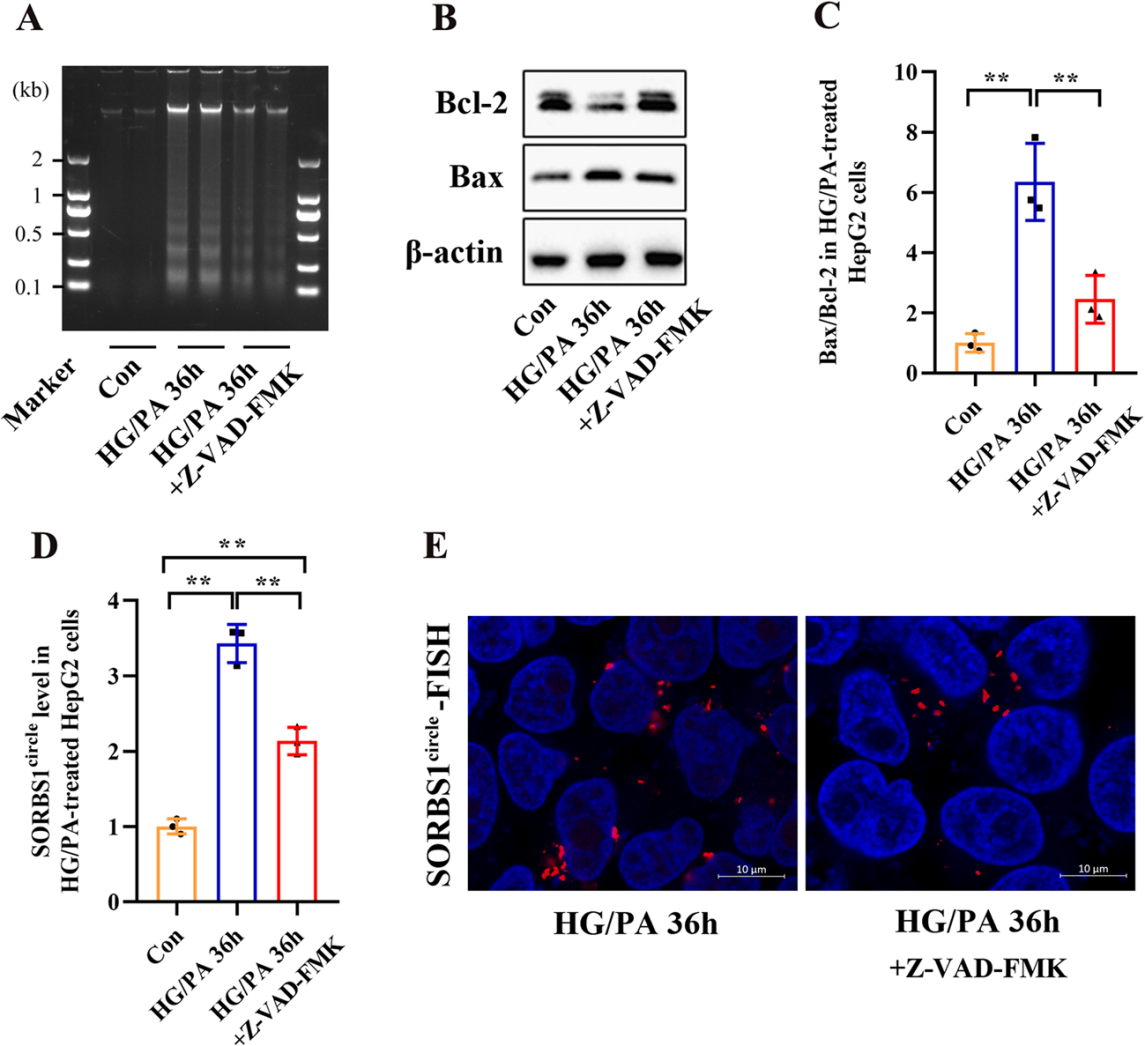
Source of eccDNA SORBS1^circle^ in HG/PA-treated HepG2 cells. **A**–**D** DNA fragmentation, Bcl-2 and Bax protein expression, and eccDNA SORBS1^circle^ level were determined in HepG2 cells stimulated with HG/PA for 36 h in the presence or absence of 100 μM Z-VAD-FMK. **E** Visualization of eccDNA SORBS1^circle^ expression in HepG2 cells stimulated with HG/PA for 36 h in the presence or absence of 100 μM Z-VAD-FMK using FISH assay. The complete image of FISH assay was shown in Additional file 1: Fig. S2. Bar = 10 μm. Data are presented as the mean ± SD of three independent experiments. *P < 0.05, **P < 0.01

eccDNAs are generated by random ligation of oligonucleosomal DNA fragments, which can be visualized as apoptotic DNA ladders in agarose gel [8]. To determine whether increased eccDNA SORBS1^circle^ level was generated by apoptosis, HepG2 cells were treated with HG/PA in the presence or absence of Z-VAD-FMK. The successful induction of apoptosis from HG/PA treatment was confirmed by the typical DNA fragments (Fig. 5A) and an increased Bax/Bcl-2 ratio (a pro-apoptotic marker, Fig. 5B, C). The increase in apoptosis level was obviously reversed by treatment with Z-VAD-FMK (Fig. 5A–C). Furthermore, Z-VAD-FMK significantly decreased eccDNA SORBS1^circle^ level in HG/PA-treated HepG2 cells (Fig. 5D). A FISH assay was performed to detect eccDNA SORBS1^circle^ using a Cy3-labeled probe. The results indicated that the level of eccDNA SORBS1^circle^ was reduced in HG/PA-treated HepG2 cells after Z-VAD-FMK treatment (Fig. 5E and Additional file 1: Fig. S2).

## Discussion

Compared with other global sequencing methods, Circle-seq is a more sensitive method for eccDNA detection [1, 2]. The closed circular structure of eccDNAs is resistant to digestion from RNases and exonucleases, thus making them more stable compared to RNAs or linear DNAs in the circulation [3]. Previous studies have reported that eccDNAs are detectable in maternal plasma, normal subjects and tumor patients plasma and serum [3, 19, 20]. The present study used Circle-seq analysis and compared the serum eccDNAs between T2DM patients and NC subjects for the first time. 598 eccDNAs were found to be upregulated, while 856 eccDNAs were downregulated in serum obtained from T2DM patients compared with NC subjects. Five eccDNAs were randomly selected for qPCR validation with outward primers, and the results confirmed the Circle-seq data to some extent.

A novel circulating eccDNA named SORBS1^circle^, the most obviously increased serum eccDNA, was identified in newly diagnosed T2DM patients. eccDNA SORBS1^circle^ was circularized by a segment of SORBS1 DNA. The host gene SORBS1 is an important adaptor protein in the signaling pathway of insulin-stimulated glucose uptake [21]. Genetic variation of SORBS1 gene is associated with glucose homeostasis of diabetes [22, 23]. Interestingly, correlation and regression analysis demonstrated that the enhanced serum eccDNA SORBS1^circle^ level of T2DM patients was positively associated with HbA1c and HOMA-IR. Insulin resistance, which is a pathological response characterized by hyperinsulinemia and impaired glucose tolerance, may be subtle during the early stages of T2DM. The results of the present study suggested that serum eccDNA SORBS1^circle^ may be a useful biomarker for predicting and monitoring insulin resistance in T2DM patients.

Insulin resistance occurs when target tissues, such as the liver, become less sensitive to the effects of insulin [24]. We further selected a hepatocyte insulin resistance model to verify the intracellular expression of eccDNA SORBS1^circle^ during the development of insulin resistance. Fluorescent signals representing eccDNA SORBS1^circle^ obtained by FISH detection were observed off the cell nuclei (chromosomes), ascertaining the extrachromosomal feature of this eccDNA. Importantly, the intracellular content of eccDNA SORBS1^circle^ in HepG2 cells gradually increased with prolonged incubation in the presence of HG/PA. Furthermore, we demonstrated that inhibiting apoptotic DNA fragmentation production through Z-VAD-FMK treatment reduced the generation of eccDNA SORBS1^circle^ in HG/PA-treated HepG2 cells. These results implied that the increased eccDNA SORBS1^circle^ level potentially associates with the development of hepatic insulin resistance, and the upregulation of eccDNA SORBS1^circle^ is dependent on generation of apoptotic DNA fragmentation.

Although we identified altered serum eccDNAs between T2DM and NC subjects, and investigated the generation and expression of eccDNA SORBS1^circle^ in HG/PA-treated HepG2 cells in this study, it is important to acknowledge that this study is preliminary and has several limitations. First, it is worth noting that the sample size for Circle-seq analysis in this study is relatively small. This small sample size may lead to an underestimation of the number of altered eccDNAs. In addition, short exonuclease V treatment time for serum DNA samples may result in incomplete linear DNA clearance. Second, the generation and source of serum eccDNA SORBS1^circle^ are unclear. Further investigation is needed to determine whether it originates from double-strand breaks, breakage-fusion-bridge, or replication slippage in the SORBS1 gene of the liver or other tissues with insulin resistance. In addition, we could not deduce whether the increased intracellular content of eccDNA SORBS1^circle^ is the cause or a subsequent result of hepatic insulin resistance. Further studies are required to provide direct evidence of the effects of eccDNA SORBS1^circle^. Unlike traditional gene products, of which the “loss-of-function” studies could achieved through siRNA/shRNA mediated knocking down or CRISRP system mediated knocking out, there is no valid methods to reduce/delete the abundance of specific eccDNA molecules. The few reported methods used for “gain-of-function” studies was not universally effective [16], which also greatly limited the functional exploration of the bulk eccDNAs. Third, our study a single-center, small-sample experiment. Multi-center and large-samples studies are needed to confirm that serum eccDNA SORBS1^circle^ level could be used as a marker for predicting and monitoring insulin resistance in T2DM patients.

## Conclusion

We identified the changes of serum eccDNAs levels between newly diagnosed T2DM patients and NC subjects. Moreover, as the most increased serum eccDNA, SORBS1^circle^ is significantly correlated with HOMA-IR in newly diagnosed T2DM patients. In addition, increased eccDNA SORBS1^circle^ level in HG/PA-treated HepG2 cells (hepatocyte insulin resistance model) is associated with apoptotic DNA fragmentation. These findings may expand the current understanding of T2DM from eccDNAs viewpoint.

### Supplementary Information

**Additional file 1: Table S1.** The distribution of eccDNAs on chromosomes. **Table S2.** List of the top 42 upregulated eccDNAs in comparison between NC subjects and newly diagnosed T2DM patients. **Table S3.** Baseline clinical characteristics of the NC and newly diagnosed T2DM subjects. **Figure S1.** The complete image of FISH assay in Fig. 4D. **Figure S2.** The complete image of FISH assay in Fig. 5E.

## Data Availability

Data will be made available on request.

## Abbreviations

eccDNAs: Extrachromosomal circular DNAs
T2DM: Type 2 diabetes mellitus
SORBS1^circle^: Sorbin and SH3‐domain‐containing‐1^circle^
HOMA-IR: Homeostasis model assessment of insulin resistance
FISH: Fluorescence in situ hybridization
