# Supplementary material for: Increased serum extrachromosomal circular DNA SORBS1circle level is associated with insulin resistance in patients with newly diagnosed type 2 diabetes mellitus
Source: Cell Mol Biol Lett. 2024 Jan 12;29:12. doi: 10.1186/s11658-023-00530-0 (PMC10785328; doi:10.1186/s11658-023-00530-0)
Supplement: Supplementary file 1 — Additional file 1: Table S1. The distribution of eccDNAs on chromosomes. Table S2. List of the top 42 upregulated eccDNAs in comparison between NC subjects and newly diagnosed T2DM patients. Table S3. Baseline clinical characteristics of the NC and newly diagnosed T2DM subjects. Figure S1. The complete image of FISH assay in Fig. 4D. Figure S2. The complete image of FISH assay in Fig. 5E. [file 11658_2023_530_MOESM1_ESM.docx]

**SUPPLEMENTARY DATA**

**The detailed steps of qPCR assay**

Outward-directed PCR primers designed in Primer 5.0. The DNA concentration was detected by a Qubit 3.0 fluorometer (Thermo Fisher, USA). All DNA samples were configured separately with the qPCR reaction system. The system configuration was as follows: 2 × Master Mix (5 µl), 10 µM forward primer (0.5 µl), 10 µM reverse primer (0.5 µl) and water added to a total volume of 8 µl. The solution was briefly centrifuged at 5000 rpm for 5 min. The 8 µl of mixture was added to each well corresponding to the 384-PCR plate. An exonuclease-treated DNA template (2 µl) was added. Place the prepared PCR plate on ice before setting up the PCR procedure. PCR was performed following these steps: 95℃, 10 min; 40 cycles (95℃, 10 seconds and 60℃, 60 seconds ). The CT values of the target eccDNA and PGEX-5X-2 were calculated and the relative expression levels of eccDNA were analyzed using the 2^-ΔΔCt^method.

**Supplementary Table 1** The distribution of eccDNAs on chromosomes

| Chromosomes | NC  （number of eccDNAs） | T2DM  （number of eccDNAs） | Increased percentage in T2DM vs. NC |
| --- | --- | --- | --- |
| chr1 | 1533 | 1846 | 20.42% |
| chr2 | 1582 | 1811 | 14.48% |
| chr3 | 1310 | 1453 | 10.92% |
| chr4 | 1178 | 1355 | 15.03% |
| chr5 | 1158 | 1385 | 19.60% |
| chr6 | 1115 | 1279 | 14.71% |
| chr7 | 1080 | 1270 | 17.59% |
| chr8 | 914 | 1046 | 14.44% |
| chr9 | 772 | 885 | 14.64% |
| chr10 | 922 | 1071 | 16.16% |
| chr11 | 857 | 1028 | 19.95% |
| chr12 | 914 | 1061 | 16.08% |
| chr13 | 641 | 670 | 4.52% |
| chr14 | 590 | 729 | 23.56% |
| chr15 | 592 | 700 | 18.24% |
| chr16 | 573 | 713 | 24.43% |
| chr17 | 663 | 828 | 24.89% |
| chr18 | 521 | 626 | 20.15% |
| chr19 | 561 | 688 | 22.64% |
| chr20 | 452 | 580 | 28.20% |
| chr21 | 243 | 290 | 19.34% |
| chr22 | 311 | 334 | 7.40% |
| chrX | 667 | 847 | 26.99% |

**Supplementary Table 2** List of the top 42 upregulated eccDNAs in comparison between NC subjects and newly diagnosed T2DM patients

| eccDNA genomic position | Length | Fold change | *P* value | Annotated gene on eccDNA |
| --- | --- | --- | --- | --- |
| chr10:97206791-97208025 | 1235 | 5.72 | 1.29 × 10^−4^ | SORBS1 |
| chr1:230789246-230789354 | 109 | 5.56 | 2.32 × 10^−4^ | COG2 |
| chr6:35754562-35766789 | 12228 | 5.51 | 2.76 × 10^−4^ | CLPS |
| chr4:190467027-190467118 | 92 | 5.36 | 4.56 × 10^−4^ | NA |
| chr14:67834238-67834389 | 152 | 5.33 | 5.01 × 10^−4^ | EIF2S1 |
| chr1:16376480-16386335 | 9856 | 5.05 | 1.30 × 10^−3^ | CLCNKB |
| chr7:25621260-25621410 | 151 | 5.05 | 1.31 × 10^−3^ | NA |
| chr4:190467005-190467096 | 92 | 5.04 | 1.33 × 10^−3^ | NA |
| chr10:38893824-38893986 | 163 | 5.01 | 1.49 × 10^−3^ | NA |
| chr2:242927549-242927991 | 443 | 4.97 | 1.66 × 10^−3^ | NA |
| chr2:218322105-218322224 | 120 | 4.97 | 1.67 × 10^−3^ | DIRC3 |
| chr7:217309-217415 | 107 | 4.95 | 1.75 × 10^−3^ | FAM20C |
| chr17:80661759-80661836 | 78 | 4.92 | 1.90 × 10^−3^ | NA |
| chr7:137407136-137407412 | 277 | 4.88 | 2.20 × 10^−3^ | DGKI |
| chr7:14009902-14010102 | 201 | 4.86 | 2.30 × 10^−3^ | ETV1 |
| chr22:45029574-45029725 | 152 | 4.86 | 2.31 × 10^−3^ | NA |
| chr18:4548601-4548751 | 151 | 4.86 | 2.34 × 10^−3^ | NA |
| chr15:70263035-70263181 | 147 | 4.79 | 3.06 × 10^−3^ | NA |
| chr20:61661127-61661230 | 104 | 4.79 | 3.09 × 10^−3^ | NA |
| chr16:17162174-17162324 | 151 | 4.75 | 3.29 × 10^−3^ | NA |
| chr1:178664858-178665207 | 350 | 4.74 | 3.56 × 10^−3^ | NA |
| chr1:113695288-113695438 | 151 | 4.73 | 3.69 × 10^−3^ | NA |
| chr10:4474521-4474696 | 176 | 4.71 | 3.91 × 10^−3^ | NA |
| chr1:64985299-64985418 | 120 | 4.65 | 5.02 × 10^−3^ | CACHD1 |
| chr7:70711082-70711232 | 151 | 4.62 | 5.48 × 10^−3^ | WBSCR17 |
| chr11:101952654-101952805 | 152 | 4.61 | 5.60 × 10^−3^ | C11orf70 |
| chr1:156858048-156858182 | 135 | 4.60 | 5.80 × 10^−3^ | NA |
| chr11:40221821-40221971 | 151 | 4.60 | 5.88 × 10^−3^ | LRRC4C |
| chr4:142611291-142611441 | 151 | 4.58 | 6.18 × 10^−3^ | IL15 |
| chr3:60810308-60810458 | 151 | 4.56 | 6.73 × 10^−3^ | FHIT |
| chr17:77921054-77921248 | 195 | 4.54 | 6.79 × 10^−3^ | TBC1D16 |
| chr7:1597134-1597278 | 145 | 4.54 | 6.90 × 10^−3^ | TMEM184A |
| chr1:877693-877843 | 151 | 4.52 | 7.09 × 10^−3^ | SAMD11 |
| chr2:228463610-228463776 | 167 | 4.52 | 7.14 × 10^−3^ | NA |
| chr19:2572410-2572560 | 151 | 4.52 | 7.53 × 10^−3^ | GNG7 |
| chr12:232323-232504 | 182 | 4.51 | 7.28 × 10^−3^ | IQSEC3 |
| chr1:221898376-221898526 | 151 | 4.51 | 7.28 × 10^−3^ | DUSP10 |
| chrX:13587719-13587869 | 151 | 4.51 | 7.34 × 10^−3^ | EGFL6 |
| chr8:3920629-3920779 | 151 | 4.50 | 7.97 × 10^−3^ | CSMD1 |
| chr16:89683977-89684145 | 169 | 4.49 | 8.08 × 10^−3^ | DPEP1 |
| chr14:26578829-26578979 | 151 | 4.47 | 9.00 × 10^−3^ | NA |
| chr16:823413-823564 | 152 | 4.44 | 9.74 × 10^−3^ | MSLNL |

**Supplementary Table 3** Baseline clinical characteristics of the NC and newly diagnosed T2DM subjects

|  | NC | T2DM |
| --- | --- | --- |
| *n* | 40 | 106 |
| Age (years) | 44.20 ± 7.00 | 41.22 ± 10.00 |
| Sex (male, %) | 55.00 | 58.50 |
| BMI (kg/m^2^) | 23.62 ± 2.74 | 26.83 ± 3.51** |
| SBP (mmHg) | 120.27 ± 12.50 | 134.71 ± 13.72** |
| DBP (mmHg) | 78.77 ± 7.74 | 85.23 ± 10.47** |
| FBG (mmol/L) | 5.28 ± 0.43 | 10.17 ± 2.65** |
| 2 hPBG (mmol/L) | NA | 17.00 ± 3.79 |
| HbA1c (%) | 5.41 ± 0.32 | 9.86 ± 2.02** |
| FIns (μIU/mL) | 8.62 ± 2.53 | 13.23 ± 6.40** |
| 2 hPIns (μIU/mL) | NA | 40.49 ± 25.57 |
| HOMA-IR | 2.04 ± 0.70 | 5.93 ± 3.27** |
| HOMA-β | 102.10 ± 36.86 | 46.99 ± 32.70** |
| TC (mmol/L) | 4.08 ± 0.59 | 5.09 ± 1.10** |
| TG (mmol/L) | 1.06 ± 0.44 | 2.77 ± 2.99** |
| HDL-C (mmol/L) | 1.34 ± 0.30 | 1.15 ± 0.21** |
| LDL-C (mmol/L) | 2.30 ± 0.51 | 2.95 ± 0.90** |
| ALT (U/L) | 16.23 ± 9.10 | 51.47 ± 43.11** |
| AST (U/L) | 18.28 ± 6.50 | 29.93 ± 18.31** |
| Cr (μmol/L) | 60.79 ± 10.79 | 57.32 ± 16.22 |
| UA (μmol/L) | 266.36 ± 58.17 | 362.33 ± 103.36** |

Data are the mean ± standard deviation (SD). ***P* < 0.01 versus normal control (NC) subjects. BMI, Body mass index; SBP, systolic blood pressure; DBP, diastolic blood pressure; FBG, fasting blood glucose; 2 hPBG, 2-hour postprandial blood glucose; HbA1c, glycosylated hemoglobin A1c; FIns, fasting insulin; 2 hPIns, 2-hour postprandial insulin; HOMA-IR, homeostasis model assessment of insulin resistance; HOMA-β, homeostasis model assessment of islet β-cell function; TC, total cholesterol; TG, triglycerides; HDL-C, high-density lipoprotein cholesterol; LDL-C, low-density lipoprotein cholesterol; ALT, alanine transaminase; AST, aspartate transaminase; Cr, creatinine; UA, uric acid.


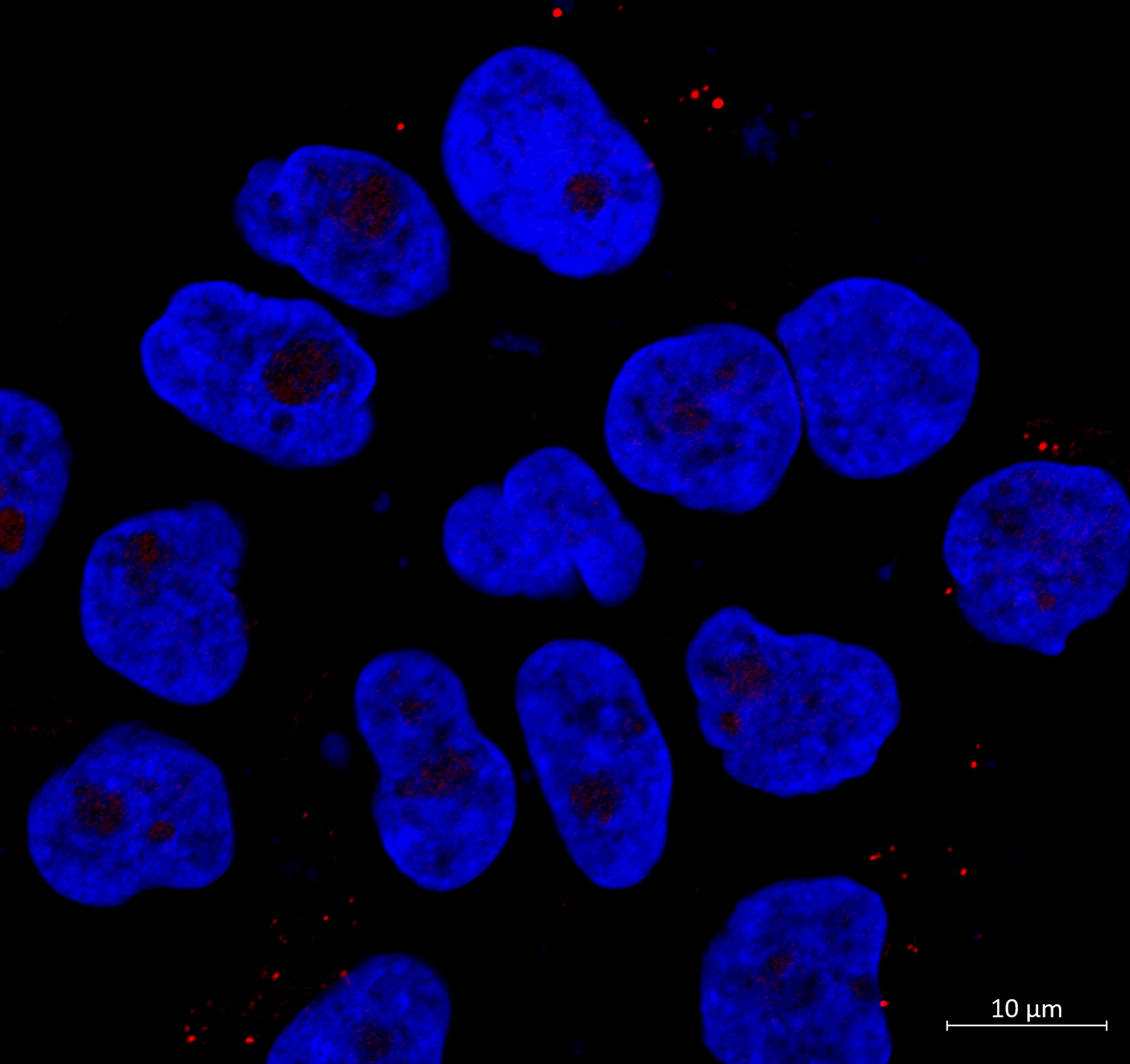


Control HepG2 cells


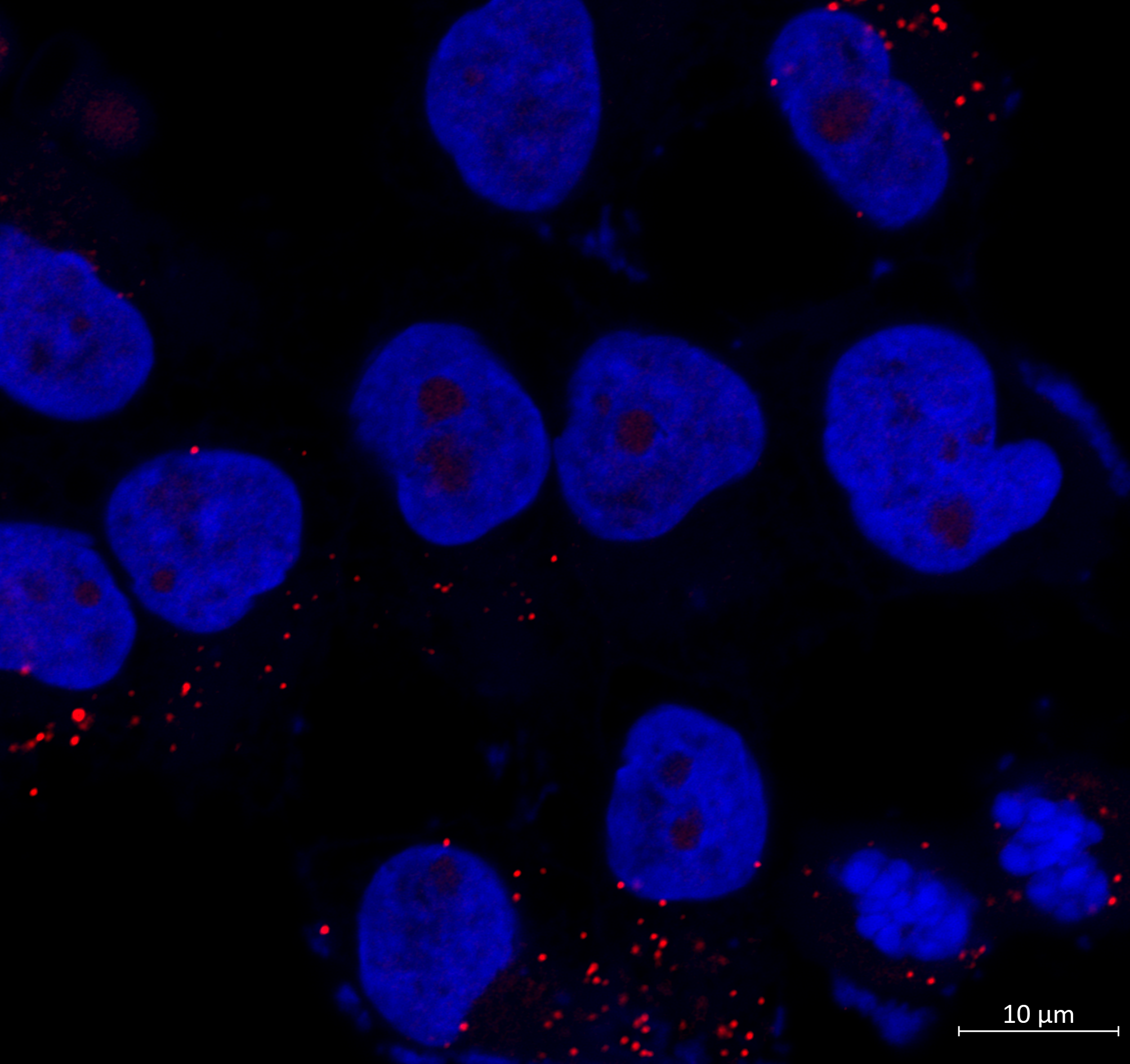


HG/PA-treated HepG2 cells (36h)

**Supplementary Fig. 1 The complete image of FISH assay in Fig. 4D.**


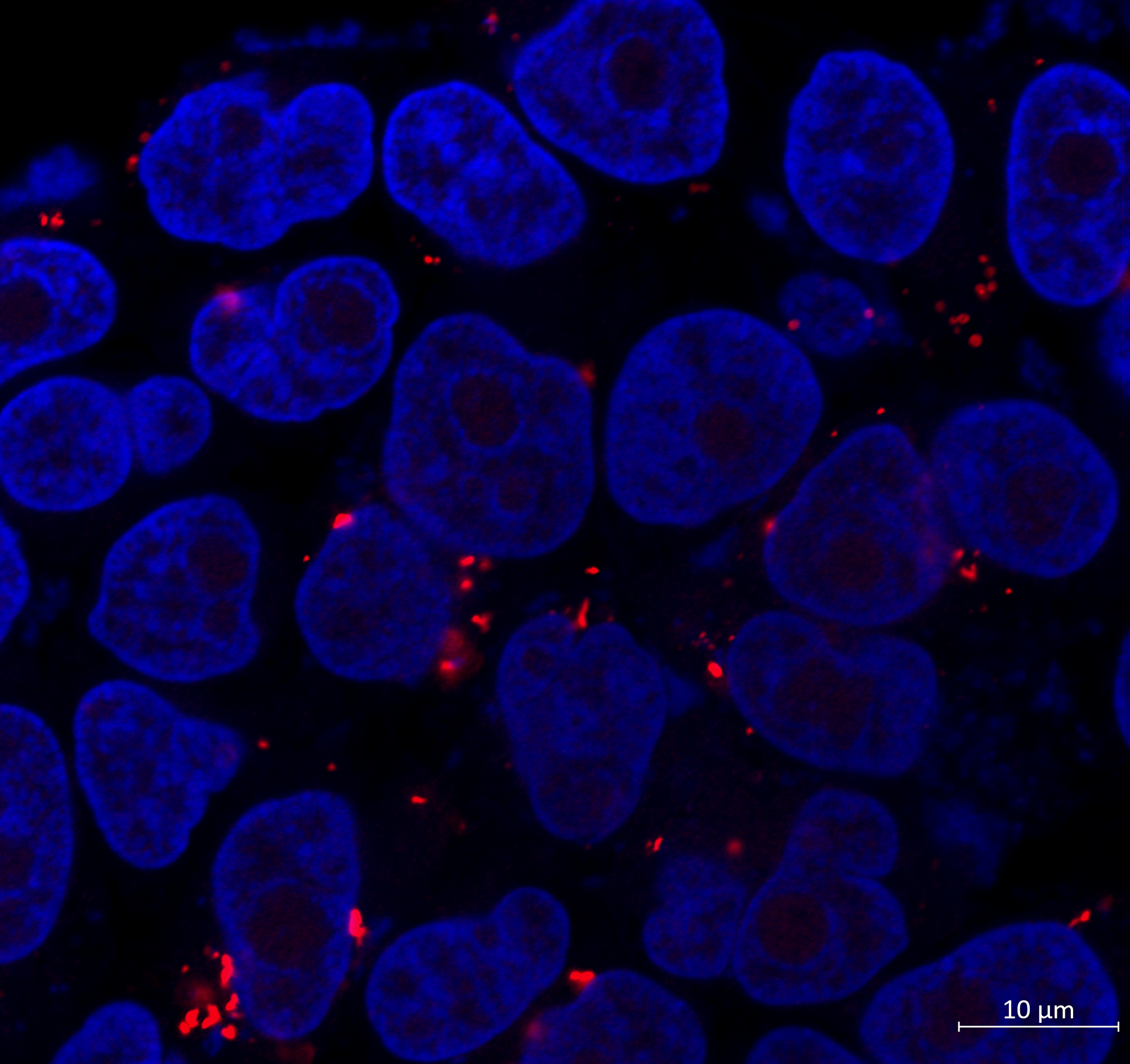


HG/PA-treated HepG2 cells (36h)


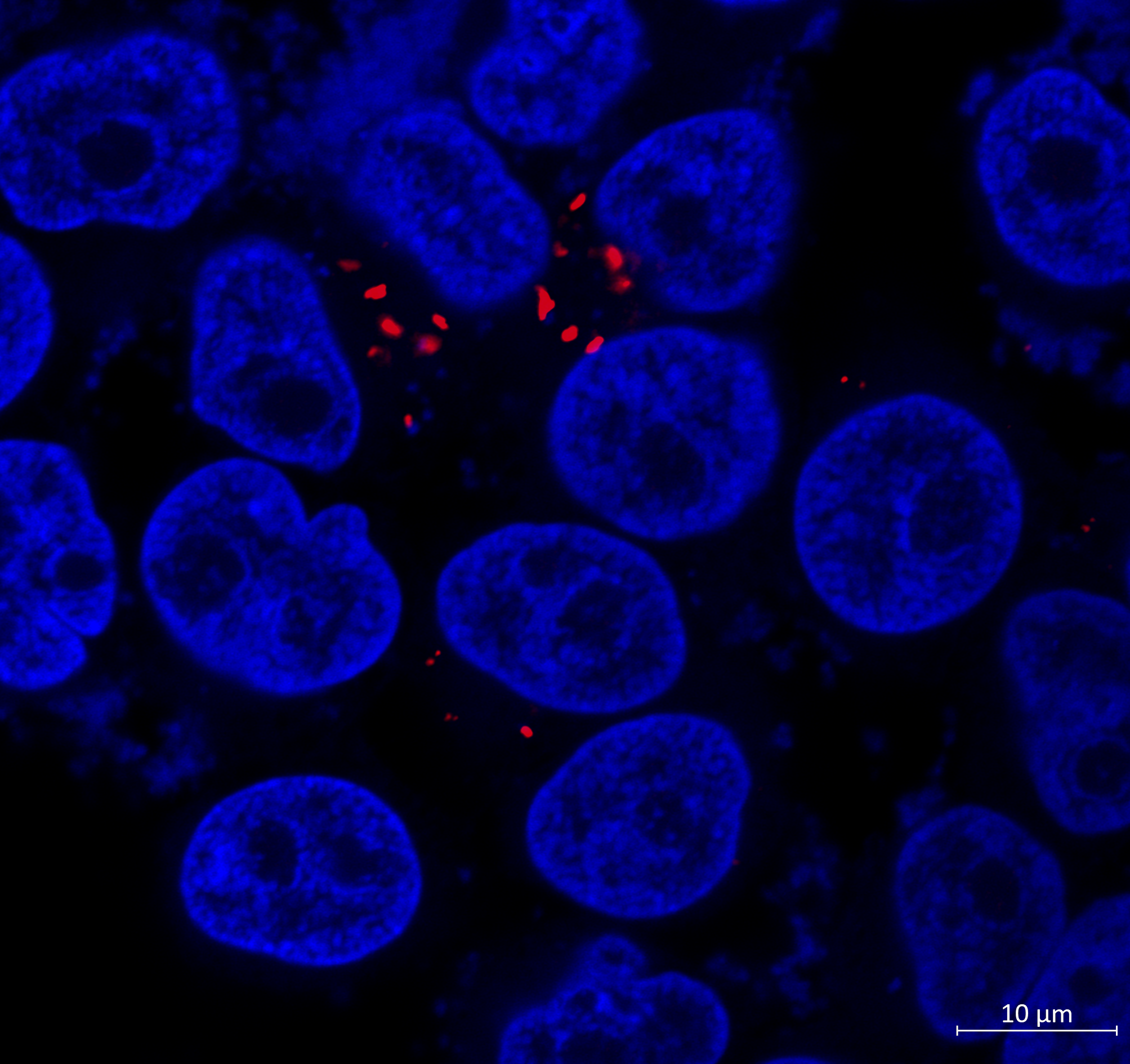


HG/PA-treated HepG2 cells (36h) with 100 μM Z-VAD-FMK

**Supplementary Fig. 2 The complete image of FISH assay in Fig. 5E.**
